# Improvements in Cycling but Not Handcycling 10 km Time Trial Performance in Habitual Caffeine Users

**DOI:** 10.3390/nu8070393

**Published:** 2016-06-25

**Authors:** Terri Graham-Paulson, Claudio Perret, Victoria Goosey-Tolfrey

**Affiliations:** 1 School of Sport, Exercise and Health Sciences, Peter Harrison Centre for Disability Sport, Loughborough University, Epinal Way, Loughborough LE113TU, UK; t.s.graham@lboro.ac.uk; 2 Swiss Paraplegic Centre, Institute of Sport Medicine, Guido A. Zäch-Strasse, Nottwil 6207, Switzerland; claudio.perret@paraplegie.ch

**Keywords:** exercise, ergogenic, upper-body, sport, supplement

## Abstract

Caffeine supplementation during whole-/lower-body exercise is well-researched, yet evidence of its effect during upper-body exercise is equivocal. The current study explored the effects of caffeine on cycling/handcycling 10 km time trial (TT) performance in habitual caffeine users. Eleven recreationally trained males (mean (SD) age 24 (4) years, body mass 85.1 (14.6) kg, cycling/handcycling peak oxygen uptake ($\overset{\cdot }{V}$_peak_) 42.9 (7.3)/27.6 (5.1) mL∙kg∙min^−1^, 160 (168) mg/day caffeine consumption) completed two maximal incremental tests and two familiarization sessions. During four subsequent visits, participants cycled/handcycled for 30 min at 65% mode-specific $\overset{\cdot }{V}$_peak_ (preload) followed by a 10 km TT following the ingestion of 4 mg∙kg^−1^ caffeine (CAF) or placebo (PLA). Caffeine significantly improved cycling (2.0 (2.0)%; 16:35 vs. 16:56 min; *p* = 0.033) but not handcycling (1.8 (3.0)%; 24:10 vs. 24:36 min; *p* = 0.153) TT performance compared to PLA. The improvement during cycling can be attributed to the increased power output during the first and last 2 km during CAF. Higher blood lactate concentration (Bla) was reported during CAF compared to PLA (*p* < 0.007) and was evident 5 min post-TT during cycling (11.2 ± 2.6 and 8.8 ± 3.2 mmol/L; *p* = 0.001) and handcycling (10.6 ± 2.5 and 9.2 ± 2.9 mmol/L; *p* = 0.006). Lower overall ratings of perceived exertion (RPE) were seen following CAF during the preload (*p* < 0.05) but not post-TT. Lower peripheral RPE were reported at 20 min during cycling and at 30 min during handcycling, and lower central RPE was seen at 30 min during cycling (*p* < 0.05). Caffeine improved cycling but not handcycling TT performance. The lack of improvement during handcycling may be due to the smaller active muscle mass, elevated (Bla) and/or participants’ training status.

## 1. Introduction

Low-moderate doses of caffeine (3–6 mg per kilogram of body weight (mg∙kg^−1^)) have been shown to positively influence cycling time-trial (TT) performance [1,2]. During cycling, the leg musculature provides the speed-generating force. However, there are numerous sports and activities such as kayaking, handcycling, double-poling and wheelchair sports during which the arms produce this force. It is apparent that nutritional supplements such as caffeine are commonly used in both able-bodied (AB) [3,4] and disability sports [5], including many that involve upper-body exercise (UBE). The physiological responses to whole- and lower-body exercise (LBE) differ to those of UBE [6], and it is therefore debatable whether the findings from the aforementioned cycling studies are transferable to an UBE sport such as handcycling.

A potential mechanism of caffeine is its influence on the central nervous system (CNS) by which it acts as an adenosine receptor (most likely A_1_ and A_2a_) antagonist [7,8]. Antagonism reduces the influence of adenosine and produces motor-activating and arousing effects. Caffeine can therefore have a positive influence on subjective feelings such as ratings of perceived exertion (RPE), mood and cognitive performance [9,10]. Lower RPE during submaximal exercise has been reported following caffeine ingestion, and/or similar RPE when a higher workload has been achieved [2,11,12]. Caffeine has also been shown to produce hypoalgesic effects during submaximal cycling in male and female participants [13,14]. It has been suggested that the inhibition of adenosine receptors following caffeine ingestion could also influence motor unit recruitment or have a direct effect on muscle [8,15]. It is likely that a combination of factors contribute to improved endurance performance but with caffeine’s influence on the CNS in mind, a similar ergogenic benefit could be expected during UBE as has been reported during LBE. However, the evidence for a positive influence of caffeine during UBE remains equivocal.

An 8 km double-poling TT performance lasting ~34 min was enhanced following the consumption of 6 mg∙kg^−1^ caffeine in regular caffeine users [12]. Double-poling is considered primarily to be an UBE; however, the trunk and legs also play a role in the performance of this technique. On the other hand, when LBE and asynchronous UBE were directly compared in very low caffeine users (<40 mg/day) during a preloaded 10 min all-out performance trial (40 min total exercise time), caffeine (5 mg∙kg^−1^) improved LBE but failed to statistically impact UBE in a mixed AB group [16]. The opposing results may be linked to differences in the exercise testing protocols, caffeine dose, training status of the participants’, or the participants’ level of habitual caffeine consumption. The contrasting responses may also be due to a number of factors related to the physiology of the leg and arm muscles. Firstly, the arms possess a smaller muscle mass and hence a reduced absolute muscle force. Arm muscles may possess a higher percentage of fast-twitch muscle fibers [17,18] and have a lower oxygen extraction capacity compared to the legs [6]. The onset of anaerobic metabolism during UBE therefore occurs at a lower level of oxygen uptake, and lactate concentrations are reported to be higher than during a comparable bout of LBE [6,19]. These factors can be altered with training however [20] and may help explain differences between performance outcomes in recreationally active participants and those that are specifically UBE trained.

It has been previously reported that caffeine increases muscular strength (maximal voluntary contraction) and motor unit recruitment in the knee extensors but not in the elbow flexors [15,16]. These observations may help to explain the lack of performance improvement during short-term UBE in AB participants [21]. The influence of caffeine on longer UBE endurance performance, however, requires further investigation given the protocols of Stadheim et al. [12] and Black et al. [16] both allowed involvement of the trunk to some extent to produce force yet report opposing effects. Black et al. [16] also used a mixed male and female participant pool of very low caffeine users, which makes their findings less applicable to the many competitive athletes who consume caffeine regularly. Therefore, the purpose of the current study was to explore the effects of caffeine on both LBE and UBE endurance performance. The study will employ an ecologically valid LBE and UBE endurance protocol whereby male habitual caffeine users will complete preloaded (30 min at 65% peak oxygen uptake ($\overset{\cdot }{V}$_peak_) 10 km TTs following the ingestion of caffeine and placebo. Importantly, they will adopt a synchronous handcycling modality for the UBE aspect, which is akin to the sports of handcycling and the cycling discipline of Para-Triathlon.

## 2. Materials and Methods 

### 2.1. Participants 

Eleven recreationally active, healthy males (age 24 (4) year, body mass 85.1 (14.6) kg, lower and upper body $\overset{\cdot }{V}$_peak_ 42.9 (7.3) and 27.6 (5.1) mL∙kg∙min^−1^) participated in the current study. Caffeine users, with average daily caffeine intake 160 (168) mg/day were recruited to represent the usual dietary habits of athletes. All procedures were approved by the Loughborough University Ethics Approvals Sub-committee (R14-P79, 10/04/14) and performed in accordance with the Declaration of Helsinki. All participants provided written informed consent and none revealed contraindications for participating in the study.

### 2.2. Experimental Design

**Figure 1:**
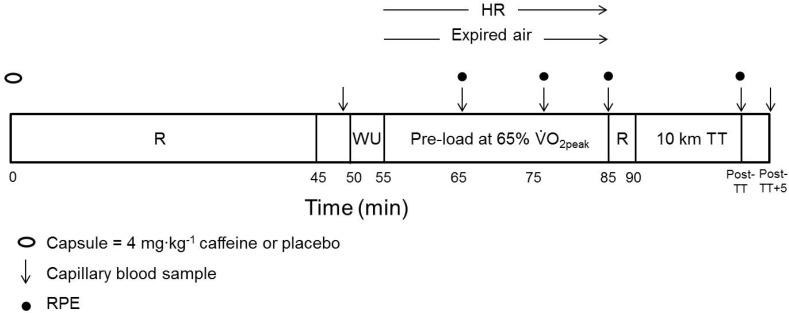
Schematic outline of the preloaded time trial (TT) experimental protocol. HR = heart rate; R = rest; WU = warm-up; and RPE = ratings of perceived exertion.

The study employed a double-blind, placebo-controlled, repeated measures design. Participants attended the laboratory on eight separate occasions, which consisted of a $\overset{\cdot }{V}$_peak_ test, a familiarization and two (caffeine and placebo) experimental trials (Figure 1) for both cycling and handcycling. Familiarization sessions aimed to limit a potential learning effect. Familiarization procedures were the same as the experimental procedures described in Figure 1 with the exception of capsule consumption and blood sampling. Experimental trials were separated by ≥48 h and were conducted at the same time of day within participants (7:30–09:30 a.m.) to avoid any influence of circadian rhythm [22].

### 2.3. Preliminary Trials

The cycling trials were performed on a Viking Jetstream 14 road bike and the handcycling trials were performed on a Draft handbike (operating in synchronous crank mode). Both pieces of equipment were mounted on a Cyclus II ergometer (Avantronic Richter, Leipzig, Germany). Bike settings were individually adjusted and standardized for each participant across trials. The differentiated RPE scale was explained to participants prior to the commencement of preliminary trial testing.

On separate occasions, participants performed incremental cycling and handcycling tests until exhaustion to determine mode-specific $\overset{\cdot }{V}$_peak_. The ergometer was set in power control mode, which ensured a pre-set power output (PO) was automatically regulated independent of cadence or gear selection by continuous adjustment of the degree of electromagnetic braking. The participants’ performed a 5-min warm-up at a self-selected pace. The continuous step tests consisted of 3-min submaximal stages with an initial load of 70 W for the cycling and 20 W for the handcycling test. Increments of 30 W for the cycling and 10 W for the handcycling test were then applied. Participants reported differentiated RPE scores at the end of each stage and upon completion. Blood lactate concentrations (Bla) were determined using a Biosen C-Line (EKF Diagnostic GmbH, Barleben, Germany) at the end of each stage from earlobe capillary blood samples. When the participant’s (Bla) increased beyond 4 mmol∙L the resistance was increased by 5 W every 15 s until volitional exhaustion (failure to maintain a cadence of ≥50 rpm following 2 warnings and an overall RPE = 19–20). Online respiratory gas analysis was carried out via a breath-by-breath system (MetaLyzer 3B, Cortex Biophysik GmbH, Leipzig, Germany). Prior to each test, gases were calibrated according to the manufacturer’s recommendations. The highest 30 s rolling average $\overset{\cdot }{V}$value was used as the participant’s $\overset{\cdot }{V}$_peak._ Heart rate (HR) was monitored continuously (Polar RS400, Polar, Kempele, Finland). 

### 2.4. Experimental Trials

Participants refrained from exercise, caffeine and alcohol consumption in the 24 h preceding each trial, as previously utilized [23]. They completed 24 h dietary diaries prior to the first experimental trial and were asked to replicate their diet for all subsequent trials. Participants were asked to consume a self-selected standardized meal 1.5 h prior to arriving at the laboratory, which was noted upon arrival (62 (10)% carbohydrate, 18 (9)% protein, 20 (9)% fat) and replicated prior to all subsequent trials. 

The experimental trials involved the consumption of either 4 mg∙kg^−1^ caffeine anhydrous (CAF) or dextrose placebo (PLA) capsules (Bulk Powders, Colchester, UK) 45 min prior to the warm-up. A 4 mg∙kg^−1^ caffeine dose has previously increased plasma caffeine concentrations to 14.6 µM, 50 min post-ingestion [23] and was therefore deemed suitable for the current study. The protocol can be seen in Figure 1 and is based on that used previously to assess the effects of glucose ingestion on UBE performance [24]. Participants were instructed to complete the 10 km TT in the shortest time possible, during which they could change gear at any time. Cycling 10 km TTs have been shown to be reproducible in active and endurance-trained participants with a coefficient of variation of 1.5% for performance time [25]. No motivation was provided during the TT and, to avoid test–retest influence, the only feedback provided was cumulative distance covered. Experimental trial conditions were temperature 19.7 (1.1) °C, pressure 1004 (11) hPa and humidity 52 (12)%. 

The 6–20 RPE scale [26] was used as a measure of perceived exertion during exercise at 10, 20 and 30 min during the preload, and post-TT. Participants were asked for three RPE scores: peripheral (muscle and joint exertion) (RPE_P_), central (ventilatory and circulatory exertion) (RPE_C_) and overall (integrated) (RPE_O_). 

### 2.5 Statistical Analyses 

Statistical Package for the Social Sciences version 20 software (SPSS Inc., Chicago, IL, USA) was used to analyze the data. Normal distribution was confirmed using the Shapiro–Wilk test and consequently (Bla) performance times, HR, power output (PO), respiratory exchange ratio (RER) and $\overset{\cdot }{V}$ data are reported as mean (standard deviation) (SD). Repeated measures analysis of variance (ANOVA) was used to examine differences in (Bla) and preload HR, RER and PO. Post-hoc paired samples t-tests using the Bonferroni correction were applied following significant findings. Ten km TT performance was also analyzed using a repeated measures two-way ANOVA, with time and treatment as within participant factors and trial order as a covariate. Cohen’s d effect sizes (ES) are included to supplement important findings. An ES of 0.2 was considered small, 0.5 moderate and 0.8 large. One-way ANOVAs with habitual caffeine intake (low, moderate, and high users) as a factor were also employed. Nonparametric ordinal RPE data are reported as median (quartiles) and were analyzed using Friedman and Wilcoxon tests. Statistical significance was accepted at *p* < 0.05. 

## 3. Results

### 3.1. Performance Tests

**Figure 2:**
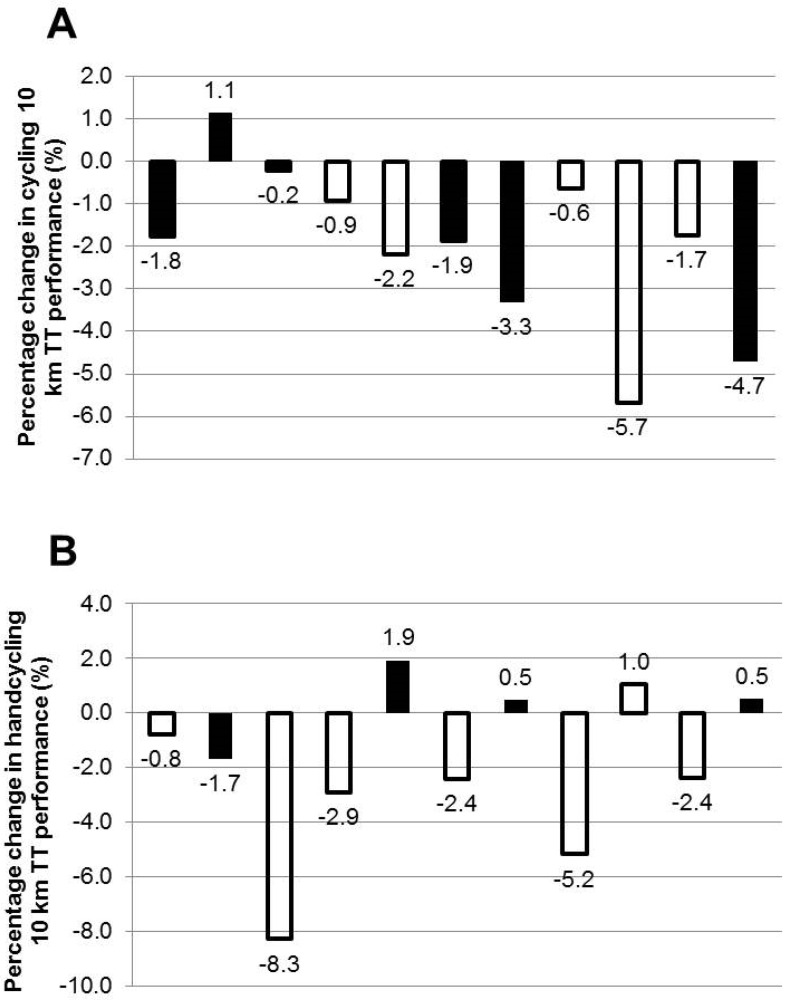
Individual percentage change in 10 km (**a**) cycling and (**b**) handcycling time trial (TT) performance. Negative responses indicate a reduction in time to complete the TT during caffeine (CAF) compared to placebo (PLA). Open/filled bars indicate participants with a $\overset{\cdot }{V}$_peak_ above/below the mode-specific mean. Participant data are ordered the same in A and B.

Caffeine significantly improved 10 km TT performance during cycling by 2.0 (2.0)% compared to PLA (ES = −0.4, *p* = 0.033) (995 (46) s and 1016 (58) s, respectively). Ten (of 11) participants cycled faster during CAF (Figure 2). Participants (7 of 11) also handcycled 1.8 (3.0)% faster during CAF compared to PLA (1450 (86) and 1476 (67) s, respectively) (Figure 2); however, this failed to reach significance (ES = −0.34, *p* = 0.153). There was no significant influence of trial order during cycling (*p* = 0.164) or handcycling (*p* = 0.298). The PO was significantly greater during CAF compared to PLA during cycling only (*p* = 0.003), and this was apparent during the first and last 2 km of the TT (*p* < 0.006). There was no influence of habitual caffeine intake on TT performance (*p* > 0.470). Participants with a handcycling $\overset{\cdot }{V}$_peak_ greater than the mean value (27.6 ml∙kg∙min^−1^) (*n* = 7) improved their handcycling TT performance by 3.2% whereas those with a $\overset{\cdot }{V}$_peak_ less than the mean (*n* = 4) had a 0.3% reduction in handcycling performance (Figure 2). 

A significantly lower $\overset{\cdot }{V}$_peak_ was recorded during handcycling compared to cycling (27.6 (5.1) and 42.9 (7.3) mL∙kg∙min^−1^, *p* = 0.001). The target relative exercise intensity of the 65% $\overset{\cdot }{V}$_peak_ during the preload was matched experimentally with average $\overset{\cdot }{V}$ values of 64.5 (2.5)% during cycling, and 59.7 (4.8)% during handcycling but importantly, did not differ between mode-specific CAF and PLA trials (*p* > 0.217). Average preload HR and RER did not differ between CAF and PLA (*p* > 0.180).

### 3.2. Blood Lactate Concentration

**Figure 3:**
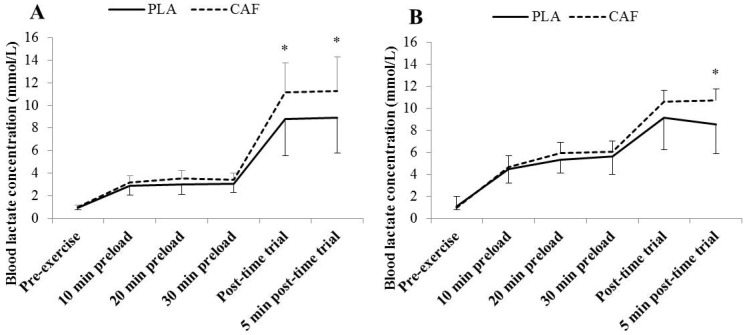
Group mean (SD) blood lactate concentrations (mmol/L) throughout the 30-min preloaded (65% $\overset{\cdot }{V}$_peak_) 10 km time trial protocol during cycling (**a**) and handcycling (**b**) following the consumption of 4 mg∙kg^−1^ caffeine (CAF) or placebo (PLA). * Significantly different from placebo (PLA).

There was a significant increase in (Bla) over time during all trials (*p* = 0.001). This was evident between 10 and 20 min during cycling following CAF only (*p* = 0.006), and at both 20 and 30 min compared to 10 min during handcycling following both CAF and PLA (*p* < 0.005). The TT resulted in a significant increase in (Bla) post-TT and five min post-TT during all trials (*p* < 0.017). The ingestion of CAF resulted in significantly higher (Bla) compared to PLA during cycling (*p* = 0.001) and handcyling (*p* = 0.007), but differences were only evident post-TT (*p* < 0.012) (Figure 3). The handcycling preload (despite a slightly lower relative workload) produced significantly greater (Bla) than during cycling regardless of trial (*p* = 0.004 and 0.016 during PLA and CAF, respectively). However, there was no difference in (Bla) pre-exercise or post-TT between modalities (*p* > 0.134).

### 3.3. Subjective Feelings

Participants’ RPE responses can be seen in Table 1. Only one participant, a low caffeine user, experienced side effects during CAF, which were reported as feelings of sickness post-preload. Only two participants correctly identified the treatment in all four trials. 

## 4. Discussion

This is the first study to assess the effect of caffeine on 10 km TT performance during both cycling and handcycling in habitual caffeine users. The main finding was that the ingestion of caffeine (4 mg∙kg^−1^) significantly improved cycling 10 km TT performance, whereas the same dose did not statistically improve handcycling performance. This study compliments the work of Black et al. [16] by investigating the influence of caffeine on longer-term endurance performance during LBE (~47 vs. 40 min) and UBE (~54 vs. 40 min) in the same habitual caffeine users. It also supports a large body of evidence on the positive impact of caffeine on endurance cycling performance [1,11,16,27].

### 4.1. Preload

The ingestion of CAF during the submaximal preload resulted in changes in RPE but not average RER, HR or $\overset{\cdot }{V}$, which agrees with earlier studies [28,29]. While there was a trend for greater (Bla) during the preload following CAF, in contrast to previous steady state exercise data [16] this did not reach significance. 

Recent reviews on caffeine and its ergogenic effects propose the antagonism of adenosine receptors as the primary mode of action leading to enhanced performance [30,31]. This mechanism of action has been shown to influence the CNS [7], through which perceived pain, effort and fatigue are reduced. The current results show caffeine to reduce RPE during constant rate LBE and UBE. During cycling, RPE_O_ was lower at all preload time-points and RPE_P_ and RPE_C_ was lower at 20 and 30 min following CAF, respectively. During handcycling, RPE_O_ was lower at 10 and 20 min and RPE_P_ was lower at 30 min only following CAF. The reduction in perceived effort during the preload may have influenced the participant’s effort during the subsequent cycling TT yet appears not to have impacted the handcycling TT. 

### 4.2. Time Trial Performance

The 10 km TT provided data from which the influence of caffeine on endurance performance could be assessed in a sport-specific manner. The ingestion of CAF resulted in a significant improvement in cycling performance (2.0 (2.0)%) compared to PLA, which was due to the increased PO during the first and last two km. On the other hand, it failed to significantly improve handcycling performance (1.8 (3.0)%) and there was large intra-individual variability. The small effect sizes (−0.4 and −0.34 for cycling and handcycling, respectively) reflect the large standard deviations for both sets of results. Individual responses to caffeine supplementation have often been attributed to differing rates of caffeine metabolism, which may in turn be linked to training status and body composition [32]. Unfortunately, the rate of caffeine absorption and metabolism were not measured in the current study. Participant three, who produced the greatest handcycling $\overset{\cdot }{V}$_peak_ value of the group, improved handcycling TT performance by 8.3% following CAF, yet only improved cycling TT performance by 0.2%. Aside from the participant displaying a learning effect or having an unexplained good/bad performance, a further explanation for some of the inter-individual variability may therefore be an individual’s training status. Despite a non-significant finding, some sports practitioners would argue that if a 1.8% improvement held true for individual elite handcyclists, caffeine could positively impact performance and ultimately influence finishing positions in a sport where winning margins are small (~0.5%) [33]. The ingestion of CAF resulted in higher post-TT (Bla) during both modes of exercise. This increase in (Bla) following the ingestion of caffeine is common in the literature during both LBE [28] and UBE [12]. The increase is understandable when seen in conjunction with improved performance such as during the current cycling trials, yet remains to be explained when a performance improvement is absent as seen during the handcycling trials. The metabolic responses to exercise differ in arm and leg muscles. Arm exercise is physiologically more stressful than leg exercise and can increase adrenaline concentration, which in turn is a potent stimulant for muscle glycogenolysis [34]. The arms also have a lower oxygen extraction capacity, which results in an earlier onset of anaerobic metabolism (~50% and 75% $\overset{\cdot }{V}$_max_ during arm and leg exercise, respectively) [6]. Hence, the greater (Bla) seen in the current study during handcycling. Accumulation of (Bla) during the handcycling TT, which was further increased during CAF may have limited the participants’ ability to improve performance.

Evidence from biopsies suggests that the triceps muscle (an important force producing muscle during synchronous handcycling) exhibits a greater proportion of type II muscle fibers than the legs (vastus lateralis) [17,18]. This may partly explain a lack of performance improvement during the endurance handcycling TT (~24 min) during which type I fibers would dominate. Furthermore, type II fibers have been shown (in vitro) to be less sensitive to caffeine compared to type I fibers [35]. Hence, performance gains may be less likely following the ingestion of caffeine during exercise which relies on the arms (with a lower proportion of type I fibers). Endurance training can improve the oxidative capacity of muscle fibers [20] and hence may help to explain the observed handcycling TT improvements following caffeine in those that had an above average mode-specific $\overset{\cdot }{V}$_peak_ (Figure 2).

Previous research suggests caffeine increases muscular strength (maximal voluntary contraction) and motor unit recruitment in the knee extensors but not in the elbow flexors [15,16]. More and larger muscles are recruited during LBE compared to UBE and hence caffeine’s influence on muscle contractility may enhance LBE performance to a greater extent. This potential mechanism is supported by the improvement in cycling but not handcycling TT performance in the current study.

Although RPE was not reduced following the cycling TT, PO was higher during CAF suggesting that participants were able to cycle at a higher PO with no change in RPE. This is in line with previous literature that has shown caffeine to increase the PO/RPE ratio during a TT [1,2]. It has previously been suggested that the limitation to maximal UBE is likely due to localized fatigue rather than central circulatory factors [36]. At the end of the handcycling preload (30 min) RPE_P_ was reduced by CAF but this reduction in perceived arm and shoulder effort did not translate to improvements in TT performance. It has been suggested that caffeine is unable to have a hypoalgesic effect during heavy-severe fixed intensity exercise [16], and the same study reported no change in RPE during a 10 min asynchronous UBE performance trial. The current study adds further evidence that the reduced RPE and hypoalgesic effects seen during submaximal synchronous UBE do not translate to improved performance during a maximal performance trial. It is likely that the nociceptive stimuli contributing to the peripheral muscle pain during handcycling may be too great for the antagonism of adenosine receptors to reduce RPE and pain, and hence are unlikely to translate to improved performance.

The $\overset{\cdot }{V}$_peak_ achieved during handcycling was 64% of that achieved during cycling (range: 52%–83%), which is lower than previously reported values (~70%) [37]. This is likely due to the training status of the current participants who were not specifically trained in either cycling or handcycling. The use of recreationally trained participants helped to limit the potential difference in performance between the cycling modalities and yet meant that participants were unfamiliar with the pacing strategies required, especially during handcycling. It is worth noting that those with a handcycling $\overset{\cdot }{V}$_peak_ above the mean improved their handcycling TT performance by 3.2%, whereas those below the mean had a 0.3% reduction (Figure 2). Hence, an individual’s training status appears to affect how they respond to caffeine during UBE. This theory is supported by improvements in swimming velocity (during which a large proportion of the force is generated by the upper-body) following the ingestion of caffeine by trained but not untrained participants [38]. The authors suggested that the intra and/or extracellular adaptations resulting from specific training are necessary to benefit from caffeine during sprint performance [38]. The current results suggest that this holds true for endurance UBE performance also.

It has been suggested that one familiarization session is sufficient for reproducible results in recreationally active individuals (cycling $\overset{\cdot }{V}$_peak_ = 3.9 compared to 3.6 L·min^−1^ in the current study) completing a preloaded cycling TT [39] but it is unknown whether this is also the case for handcycling. That said, there was no statistical evidence of a trial order effect on cycling or handcycling performance, which suggests that the results cannot be solely attributed to a learning effect.

## 5. Conclusions 

Pre-exercise ingestion of caffeine (4 mg∙kg^−1^) significantly improved cycling 10 km TT performance but there was no statistical improvement in handcycling in habitual caffeine users. The positive effects of caffeine on cycling performance may be related to reductions in RPE during the preload. The lack of a statistical improvement during handcycling is possibly due to elevated (Bla) owing to both the mode of exercise and the ingestion of CAF. Furthermore, participants’ training status appears to influence the ability of caffeine to improve UBE performance. 

## Figures and Tables

**Table 1:** Overall, central and peripheral ratings of perceived exertion (RPE) at 10, 20 and 30 min during the preload and immediately post-time trial.

		Preload 10 min	Preload 20 min	Preload 30 min	Post-Time Trial
Overall RPE	C PLA	13 (12, 13)	13 (13, 14) ^†^	14 (13, 14) ^†,‡^	19 (17, 20) ^†,‡,#^
	C CAF	12 (11, 13) *	13 (12, 14) ^†,^*	13 (12, 14) ^†,^*	19 (18, 20) ^†,‡,#^
	HC PLA	13 (12, 14)	14 (12, 15) ^†^	14 (13, 16) ^†,‡^	19 (18, 20) ^†,‡,#^
	HC CAF	12 (11, 13) *	13 (12, 14) ^†,^*	14 (12, 15) ^†^	19 (18, 20) ^†,‡,#^
Central RPE	C PLA	12 (11, 13)	12 (11, 13) ^†^	13 (11, 14) ^†,‡^	18 (17, 20) ^†,‡,#^
	C CAF	12 (11, 13)	13 (12, 14) ^†^	13 (12, 14)*^,†,‡^	19 (18, 20) ^†,‡,#^
	HC PLA	12 (11, 13)	12 (11, 13) ^†^	13 (12, 14) ^†,‡^	17 (16, 18) ^†,‡,#^
	HC CAF	11 (11, 12)	13 (11, 13) ^†^	13 (11, 14) ^†^	17 (17, 19) ^†,‡,#^
Peripheral RPE	C PLA	13 (12, 13)	13 (13, 15) ^†^	14 (13, 16) ^†,‡^	19 (18, 20) ^†,‡,#^
	C CAF	13 (11, 13)	13 (12, 14) *^,†^	14 (13, 15) ^†,‡^	19 (17, 20) ^†,‡,#^
	HC PLA	14 (13, 15)	15 (13, 16) ^†^	15 (13, 16) ^†,‡^	19 (19, 20) ^†,‡,#^
	HC CAF	13 (11, 14)	14 (12, 15)	15 (12, 16) *^,†^	19 (18, 20) ^†,‡,#^

**Note:** Data are median (quartiles). * Significantly different from placebo (PLA), ^†^ significantly different from Preload 10 min, ^‡^ significantly different from Preload 20 min and ^#^ significantly different from Preload 30 min (*p* < 0.05).

## Abbreviations

The following abbreviations are used in this manuscript:

AB	Able-bodied
(Bla)	Blood lactate concentration
CAF	Caffeine
CNS	Central nervous system
HR	Heart rate
LBE	Lower-body exercise
PO	Power output
PLA	Placebo
RPE	Rating of perceived exertion
RPE_C_	Central rating of perceived exertion
RPE_O_	Overall rating of perceived exertion
RPE_P_	Peripheral rating of perceived exertion
SD	Standard deviation
TT	Time trial
UBE	Upper-body exercise
$\overset{\cdot }{V}$	Oxygen uptake
$\overset{\cdot }{V}$_peak_	Peak oxygen uptake
